# Impact of Sulfuric Acid Treatment of Halloysite on Physico-Chemic Property Modification

**DOI:** 10.3390/ma9080620

**Published:** 2016-07-26

**Authors:** Tayser Sumer Gaaz, Abu Bakar Sulong, Abdul Amir H. Kadhum, Mohamed H. Nassir, Ahmed A. Al-Amiery

**Affiliations:** 1 Department of Mechanical & Materials Engineering, Faculty of Engineering & Built Environment, University Kebangsaan Malaysia, Bangi, Selangor 43600, Malaysia; 2 Department of Machinery Equipment Engineering Techniques, Technical College Al-Musaib, Al-Furat Al-Awsat Technical University, Al-Musaib, Babil 51009, Iraq; 3 Department of Chemical & Process Engineering, Faculty of Engineering & Built Environment, Universiti Kebangsaan Malaysia, Bangi, Selangor 43600, Malaysia; amir@eng.ukm.my (A.A.H.K.); dr.ahmed1975@gmail.com (A.A.A.-A.); 4 Program of Chemical Engineering, Taylor’s University-Lakeside Campus, Subang Jaya, Selangor 47500, Malaysia; mohamedh.nassir@taylors.edu.my

**Keywords:** halloysite nanotubes, sulfuric acid, morphology, surface area

## Abstract

Halloysite (HNT) is treated with sulfuric acid and the physico-chemical properties of its morphology, surface activity, physical and chemical properties have been investigated when HNT is exposed to sulfuric acid with treatment periods of 1 h (H1), 3 h (H3), 8 h (H8), and 21 h (H21). The significance of this and similar work lies in the importance of using HNT as a functional material in nanocomposites. The chemical structure was characterized by Fourier transform infrared spectroscopy (FTIR). The spectrum demonstrates that the hydroxyl groups were active for grafting modification using sulfuric acid, promoting a promising potential use for halloysite in ceramic applications as filler for novel clay-polymer nanocomposites. From the X-ray diffraction (XRD) spectrum, it can be seen that the sulfuric acid breaks down the HNT crystal structure and alters it into amorphous silica. In addition, the FESEM images reveal that the sulfuric acid treatment dissolves the AlO_6_ octahedral layers and induces the disintegration of SiO_4_ tetrahedral layers, resulting in porous nanorods. The Bruncher-Emmett-Teller (BET) surface area and total pore volume of HNTs showed an increase. The reaction of the acid with both the outer and inner surfaces of the nanotubes causes the AlO_6_ octahedral layers to dissolve, which leads to the breakdown and collapse of the tetrahedral layers of SiO_4_. The multi-fold results presented in this paper serve as a guide for further HNT functional treatment for producing new and advanced nanocomposites.

## 1. Introduction

**Figure 1:**
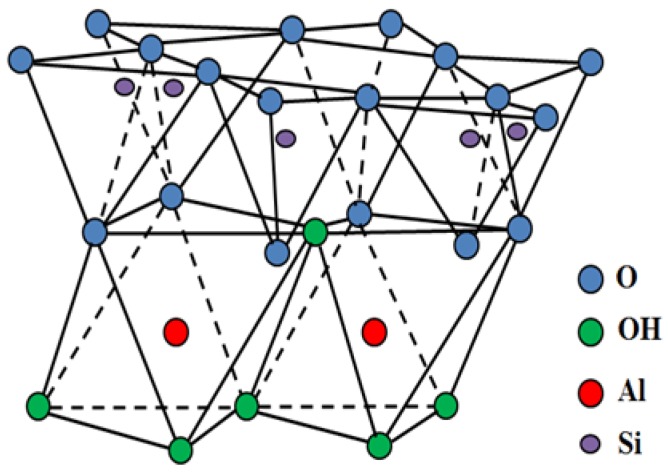
HNT molecular structure.

Aluminosilicate (Al_2_Si_2_O_5_(OH)_4_·nH_2_O), commercially known as halloysite nanotubes (HNTs), is quarried naturally from many countries around the world such as Japan, China, America, South Korea, Brazil, France, and Turkey [1]. HNT is a hollow tube-shaped-micro-to sub-micro size structure with limited high aspect ratio. HNTs are characterized by nH_2_O, whereby n is two for halloysite-7 Å and n is four for halloysite-10 Å [2]. HNT-physical characteristics are 50 to 70 nm, 15 nm, and 1.5 μm in external diameter, internal diameter, and lumen length, respectively. The basal (d_001_) spacing for halloysite-7 Å is the same as for kaolinite-7 Å. However, the basal (d_001_) spacing (7 Å) is lower than that of hydrated halloysite-10 Å. This is because the interlayer water in halloysite-10 Å evaporates, converting it into halloysite-7 Å. The crystal structure shown in Figure 1 depicts the two-layer HNT structure, and formed the tertrahedral-SiO_4_, octahedral-AlO_6_ octahedral layer. Figure 1 also shows possible bonds along with other characteristics. The water molecule interlayer separates the two layers [3,4,5].

Generally, HNT is widely used in a range of applications in numerous areas such as thermoplastic, plastic, polymer and other composites as additive fillers. In addition, its hollow nano-tubular structure makes it a potential substance for use in the production of biomedical applications [6,7]. However, the interlayer water often causes a mismatch of the tetrahedral and octahedral layer, especially at low temperatures. This causes changes in chemical and physical properties of HNTs such as cation exchange capacity [6,8,9]. As a nanoclay mineral, halloysite is used in the production of high-quality porcelain products [10] and subjected to numerous research which focuses on how HNT properties change under acid and/or heating treatment. Various methods such as acid activation, intercalation, thermal-chemical treatment and chemical modification are employed in the functionalization of HNT. All of these methods have improved the properties of HNT as well as the performances of related products. Hence, HNT has attracted considerable interest among stakeholders. It is clear that acid treatment causes disaggregation of HNT and even dissolution of the inner layers [11]. In the same study [11], the use of sulfuric acid for selective etching of alumina from the inner wall could enlarge the lumen of HNT. The effects of hydrochloric acid (HCl) have been studied thoroughly by Belver et al. [12], who focused on alkaline activation of kaolinite. At 6M-HCl, the team found that HCl treatment after 6 h results in the removal of about 90% of the octahedral Al^3+^ cations, the process that leads to creating amorphous silica with high surface area. One of the differences lies in the regular crystal structure of kaolinite that requires preheating preparation for its reaction with HCl acid [12]. In addition to HCl, sulfuric acid treatment has been employed as a traditional chemical activation method for improving the performance of nanoclay minerals [13,14,15]. The other acid used for treating HNT and kaolinite, an isomeric mineral of HNT, is sulfuric acid (H_2_SO_4_). The sulfuric acid treatment triggers several processes such as disaggregation of nanoclay particles, elimination of mineral impurities, and dissolution of the external layers. The main purpose of these processes is to break down the structure of clay minerals, resulting in an increase in surface activity [16] and BET specific surface area [12,17]. Meanwhile, Panda et al. [17] investigated the enhancement of physical and chemical characteristics of kaolin using sulfuric acid treatment. They studied the use of sulfuric acid treatment, in particular, as an effective process for producing less porous nanomaterials with high surface area [17]. One important characteristic of HNTs is that the chemistry of the outer surface of the monolayer is different from the inner surface, which means that the two surfaces have separate modifications [18]. Despite all of these preparations, there are deficiencies in HNTs such as mismatching of the tetrahedral layer and octahedral layer due to the interlayer water. When this happens, there is a direct reduction with acid. Secondly, HNTs react with acid easily due to its nanotubular structure in contrast with kaolinite that does not easily react with acid due to its plate structure [19]. Kaolinite, which shares the same chemical family as HNT, along with HNT, has almost the same benefits due to attaining the adsorption equilibrium much faster with higher pH conditions [20,21,22,23]. Regarding kaolinite, there is a variety of research that uses acids such as the sulfuric acid for treatment. One of the studies by Lenarda et al. [24] used the sulfuric acid treatment on metakaolin for preparing mesoporous catalysts [24]. The effects of using sulfuric acid on HNT physico-mechanical properties were studied by Zhang et al [2]. In their report, HNT was treated with sulfuric acid according to the specific period of times ranges between 1 and 21 h. The structure, morphology and surface characteristics of HNT were thoroughly investigated. TEM images have been shown to dissolve [AlO_6_] octahedral layers and rupture [SiO_4_] tetrahedral layers, both of which cause porous nanomaterial. The report showed that BET surface area and pore volume increase until 13 h treatment and then decrease beyond that. In addition, Zhang et al. [2] found that the micropores remain intact as far as the crystalline structure exists while the mesopores are enlarged at the time of the treatment increases. In this study, FTIR, TEM, and FESEM were utilized and a thorough investigation to surface morphology, chemical composition, and crystal structure was performed. Despite the fact that the two studies agreed on the destruction of HNT crystal structure due to sulfuric acid treatment, there are some conflicting results about the BET surface area and the number of the XRD peaks attained.

## 2. Results and Discussion

### 2.1. FTIR

**Figure 2:**
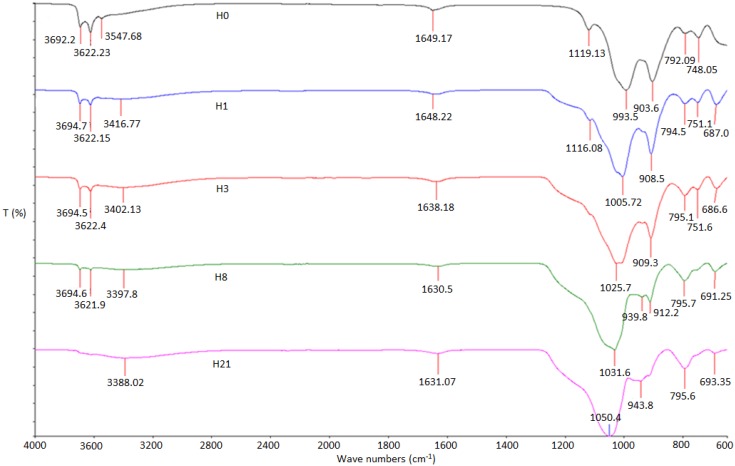
FTIR pattern of H0, H1, H3, H8, and H21 samples of HNT-sulfuric acid treatment.

Figure 2 shows the vibrational modes of FTIR spectra of treated HNTs that were assigned according to numerous previous and similar studies [25,26,27,28], and the results are tabulated in Table 1. The spectra of [O–H] stretching of inner-surface hydroxyl of H0, H1, H3 and H8 show almost similar absorption spectra at 3692.2, 3694.7, 3694.5 and 3694.6 cm^−1^, respectively [20]. In addition, the spectra of H0, H1, H3 and H8 in the [O–H] stretching of inner hydroxyl group show similar spectra absorption at 3622.23, 3622.15, 3622.4 and 3621.9 cm^−1^ [29]. The spectrum of the inner-surface hydroxyl group and inner hydroxyl group of H21 sample disappear. One possible reason for the disappearance of these two spectra is the breakage of their bonds due to longevity of the acid treatment for 21 h. The absorption spectra of [O–H] group with intermolecular hydrogen of H0, H1, H3, H8, and H21 are recorded at 3547.68, 3416.77, 3402.13, 3397.8 and 3388.02 cm^−1^, respectively [20].

The absorption spectra for H0, H1, H3, H8 and H21 recorded at 1649.17, 1648.22, 1638.18, 1630.5 and 1631.07 cm^−1^, respectively, show very weak peaks that could not be reliable to be assigned to any possible bond and might attributed to some impurities. The bonds at 1119.13 and 1116.08 cm^−1^ peaks of samples H0 and H1 are assigned to the stretching mode of apical [Si–OH] organic groups [29]. The [Si–OH] spectra disappear for samples H3, H8, and H21, suggesting that [OH] radical is no longer attached to Si due to the influence of the sulfuric acid treatment. The bands at 993.5 and 1005.72 cm^−1^ are caused by the stretching vibrations of [Si–O–Si], which is closer to the outer surface of the HNT molecules [16,29]. The strong characteristic peaks at 1025, 1031.6 and 1050.4 cm^−1^ in the HNT spectrum are assigned to the stretching vibration of [Si–O] bonds on HNT surfaces [20]. The bands at 903.6, 908.5, 909.3, 912.2, and 943.8 cm^−1^ are assigned to bending vibration of [Al–OH] [20]. The bonds at (792.09, 748.05), (794.5, 751.1), (795.1, 751.6), (795.7, 795.6) cm^−1^ peaks of samples H0, H1, H3, H8, and H21 are assigned to the stretching mode of Al-O-OH. The bonds at 687.0, 686.6, 691.25 and 693.35 cm^−1^ peaks of samples H1, H3, H8, and H21 are assigned to the stretching mode of apical alcohol-OH out of plane bend [30].

### 2.2. XRD

**Figure 3:**
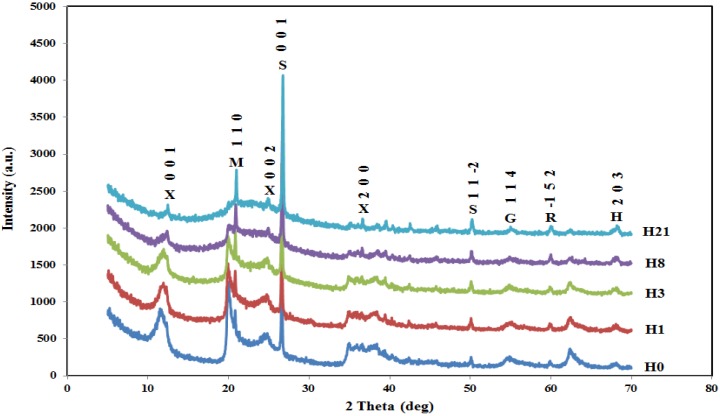
XRD spectra of H0, H1, H3, H8, and H21. X (halloysite-7 Å), M (aluminium silicon oxide), S (quartz), R (silicon oxide), G (graphite).

XRD is one of most efficient techniques used to study crystal structures. The samples of H0, H1, H3, H8, and H21 are studied using XRD as shown in Figure 3. XRD results are very similar to those reported by Zhang et al. [2] with very limited exceptions. As shown in Figure 3, the diffraction angle (2θ) was taken up to 70° compared to about 50° in Zhang’s work. The extra diffraction angle, about 20°, covers more reflections that result in a better evaluation of the effect of sulfuric acid on the crystal structure of HNT. The neat HNT shows a relatively sharp peak at 2θ of 12.80° with corresponding d value of 10.04 Å, which is the characteristic (001) peak of halloysite-(10 Å). The peak assigned for (001) does not show any shifting, suggesting no interaction of sulphuric acid with interlayer space [31]. Another peak appears at 2θ of 20.56°, which corresponds to d110=10 Å and can be ascribed to halloysite-7 Å. The next reflection appears at 2θ of 25.24°, which is assigned to (002). There is another peak at 2θ of 35.03° (011), 38.27° (200); 49.93° (11-2), and 62.62° (114). It is observed that the peak intensity at 35.03° (011) decreases as acid treatment time increases due to delamination [32]. The intensities of the peaks at 49.93° (11-2) and 62.62° (300) increase due to a greater presence of aluminum sulfate caused by the acid treatment [33]. As sulfuric acid treatment takes place for H1, H3, H8, and H4, the intensity of the peaks at a reflection of (001), (110), and (002) decreases for H1 and H3 and disappears for H8 and H21. For all other reflections, the intensity of the peaks decreases but seemingly survives at the highest treatment of H21.

### 2.3. TEM

**Figure 4:**
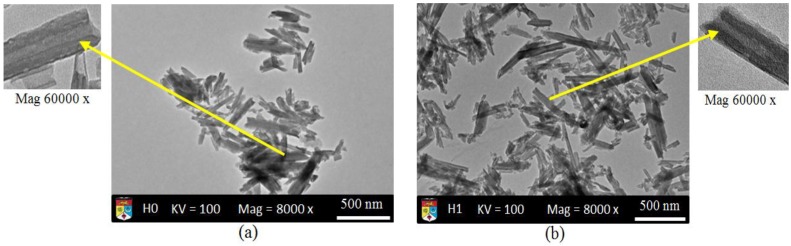

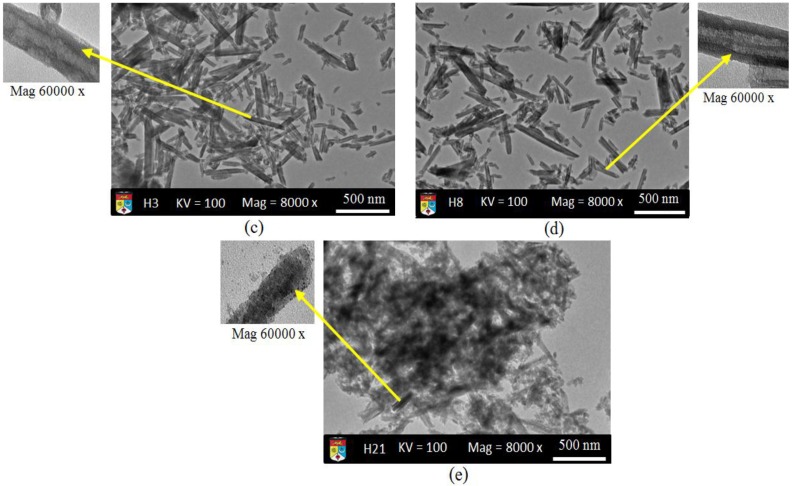
TEM images of HNT-H0 (**a**); HNT-H1 (**b**); HNT-H3 (**c**); HNT-H8 (**d**) and HNT-H21 (**e**).

TEM images of neat H0-HNT shows that HNT nanotubes are very clear with dimensions of 200–1000 nm in length, 10–50 nm in inner diameter, and 80–150 nm in outer diameter. The wall of the nanotube is composed by about 6–8 molecular layers [34]. It is assumed that the neat HNT exhibited alumite plates [3]. Figure 4b shows H1-HNT nanotubes just started to scatter due to the effect of the sulfuric acid while the single nanotubes are exfoliated on the outer surface. As the time of sulfuric acid treatment increases to 3 h (H3-HNT), the nanotubes show better distribution as indicative that more time treatment results in better HNT distribution. However, the outer surface shows clear exfoliation as depicted in Figure 4c. After 8 h of acid treatment, the nanotubes are almost detached from each other with very little agglomeration, while the outer surface of the nanotubes looks severely exfoliated as shown in Figure 4d. The nanotubes lost their physical appearance by showing no complete tube due to the severe exfoliation as shown in Figure 4e. The effect of the sulfuric acid is clearly shown on the HNT-nanotubes. The results suggest that the distribution of the nanotubes is the best distribution concerning the sequence of treatments employed in this work. It is important to note that the effectiveness of the mixing of these HNTs in composites needs more work to determine the mechanical properties.

### 2.4. FESEM

**Figure 5:**
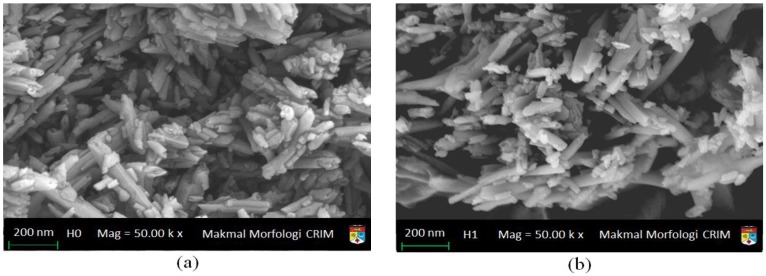

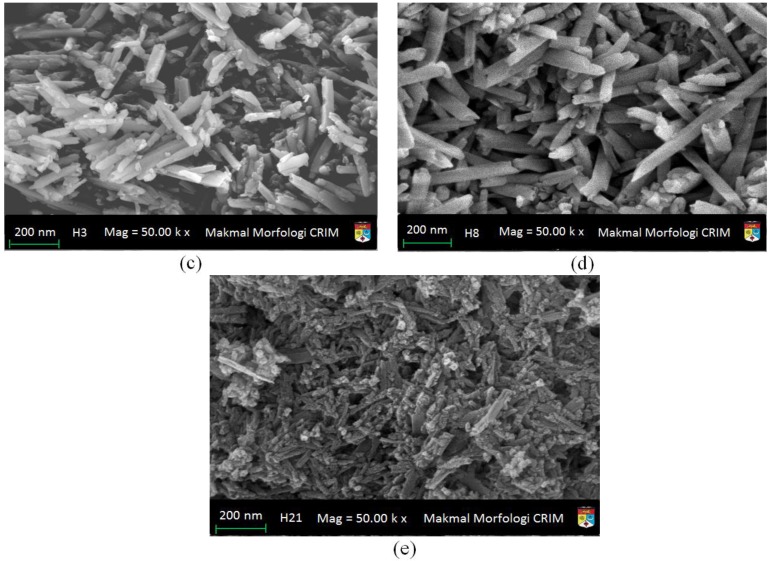
FESEM microphotographs of (**a**) H0 (Neat HNT); (**b**) H1 (acid treated of HNT for 1 h); (**c**) H3 (acid treated of HNT for 3 h); (**d**) H8 (acid treated of HNT for 8 h) and (**e**) H21 (acid treated of HNT for 21 h).

Figure 5 shows the FESEM images of HNT before (H0) and after acid treatment (H1, H3, H8, and H21). The focus in these figures is about the distribution of the nanotubes before and after sulfuric acid treatments. Considering the nature of FESEM images that signifies the distribution of the nanotubes with the virtually unlimited depth of field in the matrix rather than horizontally on the surface, the images did not clearly show the exfoliation of the outer surfaces. The stacking of HNT nanotubes clearly shows the effect of sulfuric acid on the distribution of HNT nanotubes. As the acid time treatment increases, the HNT nanotubes became separable until the 21 h HNT treatment where the HNT nanotubes appeared very mixed with no distinguishable physical feature of the nanotubes themselves. The result of 21 h-acid treatment agrees with the finding of XRD for the same sample as shown in Figure 3, where most of the reflections caused by insider planes have almost disappeared. The FESEM results for the same sample, H21, are in agreement with TEM images of the same sample as shown in Figure 4e, which shows how the outer surface is affected by this long-time treatment.

### 2.5. Mapping with EDS 

**Figure 6:**
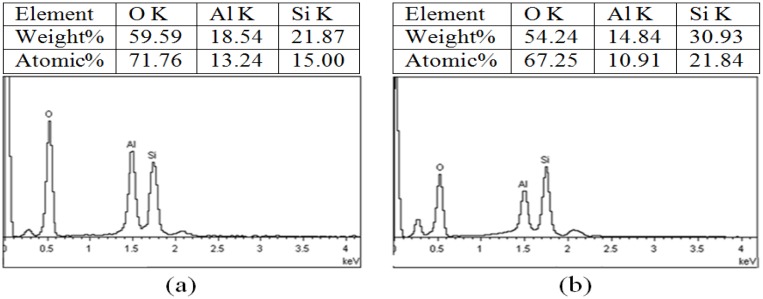

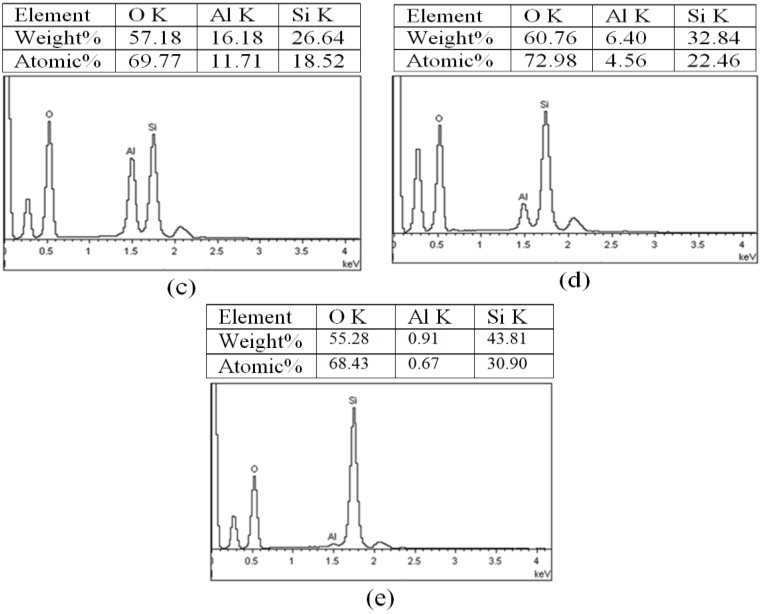
Mapping and EDX for: (**a**) (H0); (**b**) H1; (**c**) H3; (**d**) H8; and (**e**) H21.

EDS, unlike XPS, provides qualitative results based on the instrument ability for collecting data which, in most cases, lies beyond certain limitations which could be accepted at reliable precision. It is expected that EDS spectra show the relative intensities of HNT (Al_2_Si_2_O_5_(OH)_4_·nH_2_O) main components of Al, Si, and O (H cannot be seen) either by weight or atomic percentage. Figure 6a–e shows the numerical relative intensities of O, Al, and Si based on weight and atomic percentages for H0, H1, H3, H8, and H21, respectively. The percentage of O content is very crucial since O is bonded to Al, Si, and H. The acid treatment has its impact on the bonding of O with other elements in the matrix. Based on the results in the inserts of Figure 5, the O content is almost stable suggesting that the O is still localized in the matrix whether it is a bind or not. The percentage of other elements depends on their orientation and localization in the matrix.

The results of H0 and H8 presented in this paper are better to be discussed in the light of similar results obtained by a previous work conducted by Zhang et al. [2] for same loading since these two loadings are the only ones presented by the two authors as described in Table 2. The results show that the oxygen percentage is almost the same in both analyses while a significant difference was found in other components. For pure HNT (H0), aluminum %weight contents increased by 126%, while for H8, the aluminum %weight content decreased by 45%. Regarding the silicon, the small increment was reported between the two works for H0, but for H8, the increment was recorded at about 11%. Assuming that the oxygen contents have almost similar results, the ratio of Si/Al for H0 decreased from 2.46 to 1.17 while for H8, the same ratio increased from 3.17 to 5.13. One possible reason for these differences is the quality of HNT used in the analysis. Zhang and his group did not identify the source of HNT and, possibly, the quality is different from the one used in this work.

### 2.6. TGA

**Figure 7:**
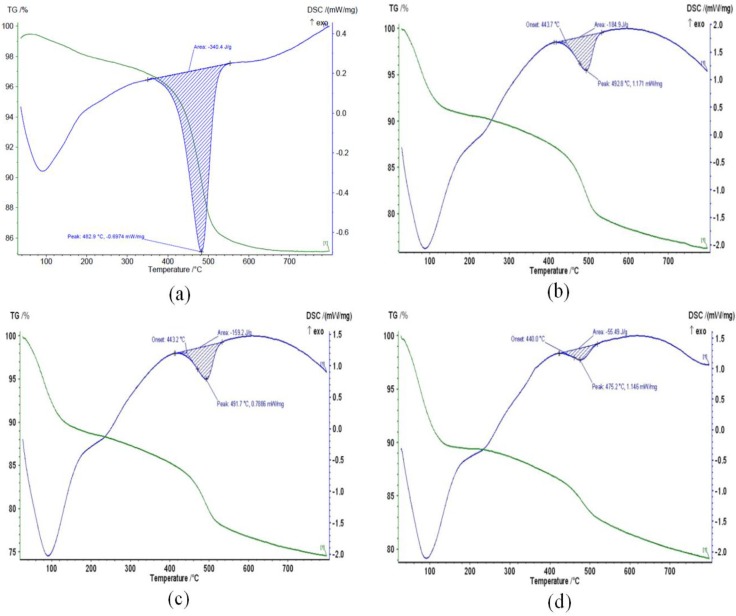

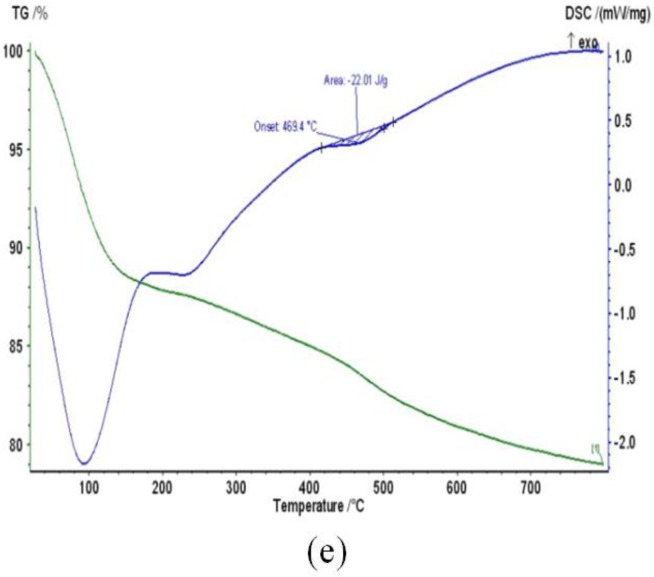
TG–DTA curves of (**a**) neat HNT (H0); (**b**–**e**) H1, H3, H8, and H21, respectively, sulfuric acid treated products as a function of the durations of sulfuric acid treatment.

The TG-DTA weight loss spectra of H0, H1, H3, H5, and H21 are shown in Figure 7. DTA spectra of the H0, H1, H3, H8, and H21 show five endothermic peaks at 482.9, 492.8, 491.7, 475.2, and 469.4 °C, respectively. For pure HNT, DTA shows the extra endothermic peak at 86 °C. The area under the curve in the figure represents the amount of heat released endothermically from the surrounding to the HNT nanotubes. For H0-HNT, the endothermic peak shifts slightly to the higher temperature from 482.9 to 492.8 °C due to acid treatment as pointed out by [13]. The shift also suggests that the crystal structure undergoes some changes. As the acid treatment reaches 3 h (H3), the endothermic peak shows no shift in temperature and the size of the peak shows no change either. As the acid time treatment increases to 8 h (H8), the peak temperature shifts towards lower temperature from 491.7 to 475.2 °C, suggesting phase change that requires an ample amount of heat to be released causing lower temperature [35]. It is noticeable that the size of the endothermic peak at 475.2 °C decreases due to the phase change. As the time of the acid treatment increases to 21 h (H21), the peak shifts again to lower temperature of 469.4 °C, suggesting the occurrence of more phase changes, which drastically reflects on the size of the endothermic peak that becomes a negligible amount, showing that the phase changes have almost been completed.

The hydroxyl groups of [O–H] (inner and outer) and [O–H] out of plane have the ability to condense and hydrate at temperatures range of 500 to 800 °C. The first stage of the water loss at low temperatures is related to the physisorbed water, whereas the high-temperature weight loss could be attributed to the dehydration and dehydroxylation of the HNT sheet. It is observed that the physisorbed water increases as the acid-treatment time increases. One possible reason for this behavior is that the sulfuric acid increases both the amount of amorphous silica and the surface area, which made the water adsorption higher. At the high-temperature side, the percentage weight loss of HNT acid treated is lower than the pure HNT that could be attributed to the removal of the octahedral Al ions along with the concurrent removal of structural hydroxyl groups [17]. For H0-HNT, the endothermic peak centered at around 86 °C may be due to physisorbed water while the peak at the higher temperature of 480 °C might be due to the liberation of water caused by dehydroxylation of coordinated and structural water molecule [36]. The higher acid concentration increased the physisorbed water and decreased the structural and coordinated water leading to change in the endothermic peaks in treated samples [17].

### 2.7. DSC

**Figure 8:**
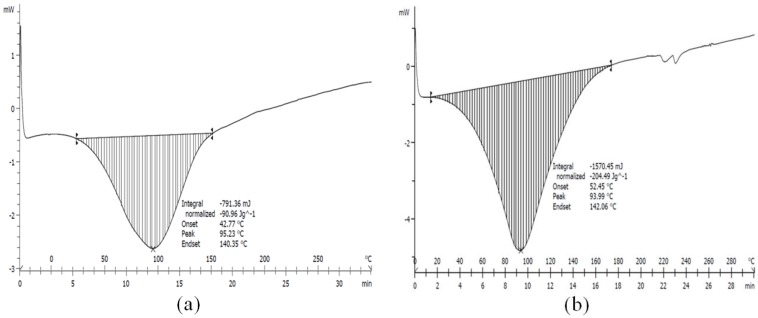

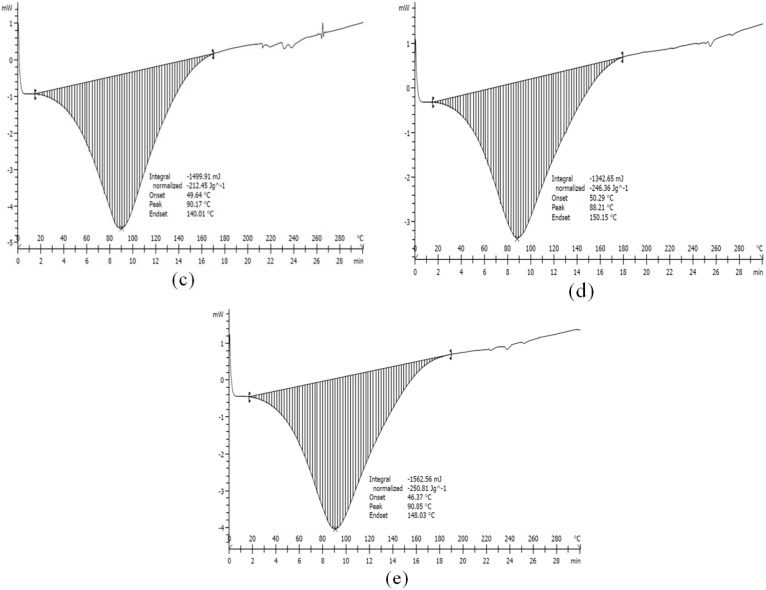
DSC curves of (**a**) neat HNT (H0); (**b**–**e**), H1, H3, H8, and H21, respectively, sulfuric acid treated products as a function of the durations of sulfuric acid treatment.

The DSC heating can be used to determine a temperature signifying the melting of the HNT. Figure 8a–e shows DSC scans for H0, H1, H3, H8, and H21, respectively. The results of DSC are summarized in Table 3. The maximum peak temperature is observed for the H0 where no chemical treatment is performed. The peak temperature is shifted from 93.95 °C to its minimum value at 88.21 °C for H8 before the peak increase to 90.85 °C for H21. The effect of the sulfuric acid on the HNT structure clearly suggests structural damaging along with exfoliation as suggested by XRD and FTIR.

### 2.8. BET

**Figure 9:**
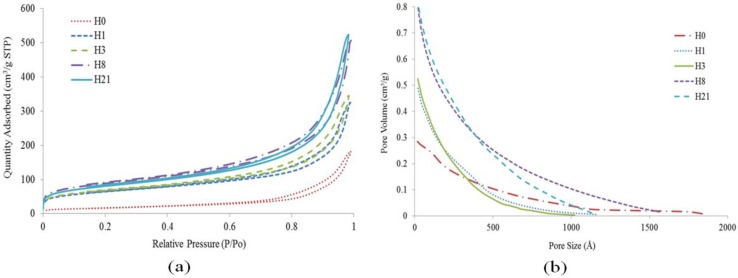
(**a**) N_2_ adsorption-desorption curves; (**b**) distribution micropore size.

The analysis of N_2_ adsorption-desorption is performed to investigate the surface area and pore volume of the neat HNT and acid-treated HNT specimens at times of 1, 3, 8, and 21 h. Table 4 tabulates the values of BET surface area and pore volume of all HNT samples-treated and untreated. All of the samples in Figure 9 display similar adsorption isotherms that are classified as type IV, which belongs to the mesoporous type according to the International Union of Pure and Applied Chemistry (IUPAC) classification [37]. The isotherm curves show a small area as a result of the adsorption and desorption curve—the area that is related to the number of N_2_ molecules adhered to the surface and not desorbed. This area becomes smaller as the acid time treatment increases, which can be explained by the TEM images shown in Figure 4e. Figure 9b shows the distributions of micropore size of natural HNT (H0) and sulfuric acid treated products (H1, H3, H8, and H21). When sulfuric acid treated time is increased from 1 to 21 h, the distribution of the micropore size of HNT shows a big difference while the pore size and pore volume indicates an increases [38]. The distribution of a fixed micropore size is to facilitate the utilization of acid treated HNT for various applications such as drug supporters, enzyme carriers, and selective adsorbents.

There is a significant increase in the BET surface area of HNT from 59.04 m^2^/g (H0) to 222.55 m^2^/g (H1). These findings are higher than those reported by Belver et al. [12] because of a high development of both the internal and the external surface of the HNT. This increase slows down as the acid time treatment moved from H1 to H3 and then to H8, where BET surface area reached the maximum of 306.43 m^2^/g. These developments are very well correlated to the dissolution of AlO_6_ octahedral layers during acid treatment as suggested by XRD spectra shown in Figure 2. As the acid time treatment increased to 21 h (H21), the BET surface area of HNT decreases to 279.5 m^2^/g possibly due to the disaggregation of silica layers. It is also noted that the micropore volume of HNT changes slightly from H1 (1 h) to H8 (8 h) due to the formation and blocking of micropores [39]. The other finding reveals that the average pore size of HNT treated by sulfuric acid shows an increase from 9.94 nm at 1 h to 11.74 nm at 21 h; however the maximum pore size of 11.74 nm is below the average pore size of natural HNT (16.73 nm) due to the opening of HNT.

The data presented in the second and the last column of Table 4 compares the current results with those obtained by Zhang et al. [2] for the BET surface area. Both results agree that a significant change in the surface area took place at or around 1 h of acid treatment (H1); however, there is no agreement between the two findings regarding accomplishing the maximum surface area. For Zhang et al. [2], the maximum appears at H3, while, for the current work, the maximum appears at H8. The two results also showed that, at H8, the result of the current work is better than the result presented by Zhang et al. [2].

Seemingly, there are some significant differences between the current work and the work presented by Zhang et al. [2]. One possible reason for these differences is the origin of the HNT nanotubes. HNTs for this work are imported and chemically checked in the USA while the origin of the halloysite used in Ref. [2] is not mentioned and is more likely a commercial grade. In addition to this, the HNT impurities, as reported by Zhang et al. [2], constitute about 4%, while the impurities in HNTs used in this work are less than 0.59%. Apparently, XRD [2] results showed more reflections than the one used in this study, possibly for high impurity percentage. It is important to note that the trend of the results presented here or by Zhang et al. [2] have the same common ground, and the possible differences could be considered secondary factors that do not significantly affect the overall findings. 

## 3. Materials and Methods 

The main materials used in this experiment are sulfuric acid and HNTs. The sulfuric acid is of purity 95%–98%, molecular weight 98.08 g/mol, and supplied by Sigma-Aldrich (Saint Louis, MO, USA). HNT was supplied by Natural Nano, Inc. (New York, NY, USA). Table 5 shows the chemical compositions and physical properties of HNTs.

### Acid Treatment

The procedure of acid treatment for HNTs is carried out as follows: add 15 g of HNT into 100 mL of 3M sulfuric acid and divide the mixture into four portions and the samples are kept in a water bath at a steady temperature of 90 °C. The four mixtures are separately stirred at a speed of 200 rpm for 1, 3, 8, and 21 h, respectively. Each mixture is centrifuged at the speed of 3000 rpm for 10 min to separate the paste from the solution. The paste was washed away using distilled water up to four times and then dried in an oven at 70 °C for 12 h. Finally, the dried HNTs were ground using a mortar. The samples were labeled as H0, H1, H3, H8 and H21 based on the duration of the sulfuric acid treatment interval [40].

## 4. Characteristics

In this section, the characterization of the HNTs treated at different times with acid are presented, which includes FTIR, XRD, TGA, DSC, TEM, FESEM, and BET. FTIR is used to identify the functional group of HNTs. The analysis is performed using a Perkin Elmer System 2000 (Waltham, MA, USA), which is equipped with attenuated total reflectance. For this research, the range of the FTIR spectrum was set between 600 and 4000 cm^−1^ at a resolution of 4 cm^−1^. Secondly, the HNT structure and crystallite size are investigated using XRD model D8 with advance Bruker AXS X-ray and Cu radiation of 1.5406 Å (Berlin, Germany). XRD is equipped with the EVA software (Version 2, Bruker Corporation, Karsruhe, Germany) to evaluate the structure and lattice strain of samples. All XRD patterns are compared for standardization with the Joint Committee on Powder Diffraction Standards (JCPDS). The third characterization is TGA. TGA is used to identify the thermal properties of all five specimens of H0, H1, H3, H8, and H21. The analysis is performed using the TGA Model Q600 of TA Instrument, New Castle, USA. The TGA tests were carried out in a nitrogen gas environment at a flow rate of 60 mL/min, while the temperature is set between 25 and 800 °C scanning rate of 10 °C/min. Another characterization is conducted using DSC (Model Q2000, TA Instrument New Castle, DE, USA), which is used to analyze the melting temperature of H0, H1, H3, H8, and H21. All DSC analyses were performed under an inert atmosphere of nitrogen flows at rate of 50 ml/min, between −20 and 250 °C, and at a scanning rate of 10 °C/min. Regarding morphological images, the field of nano-science considers TEM as the most important instrument for studying the samples morphology and particle size. TEM of Philips, model CM12 (Somerset, NJ, USA), is operated at 80 kV, which generates an electron beam capable of passing through the specimen and interacts with it, producing an image that serves to study the surface and the particle size. The sample is prepared by dispersing the proper amount of the powder in 10 mL ethanol using the ultrasonic water bath for 10 min. The images are then magnified and directed to a fluorescent screen for further investigation. In addition to TEM, FESEM of model Zeiss SUPRA 55-VP (Konigsallee, Deutschland) is used to investigate and view the morphology of the HNT samples. FESEM is equipped with a higher resolution, and it has lower charging on the sample surface. The magnification of the morphology observations is set at 200 k. The elemental analysis of HNT is performed using OXFORD EDS and Mapping (Version 2, Omniprobe, Dallas, TX, USA). The isotherm technique of accuracy of ±0.02 m^2^/g has been used a technique known as BET for a quite long time. BET is used to determine the surface area of the materials by calculating the physical adsorption of nitrogen gas molecules. The surface area of the sample is analyzed using a Gemini apparatus (Micrometrics ASAP 2020, Norcross, GA, USA). The BET analysis requires degassing the sample at certain temperature, time, and vacuum. In this study, the degassing process is carried out at 350 °C for 2 h under the vacuum of 50 mTorr. Using suitable software, the Barrett–Joyner–Halenda (BJH) equation is used to calculate the pore volume as well as the average pore size of the distribution by exploiting the nitrogen desorption isotherm [41]. The total surface area of the sample can be determined by the number of nitrogen molecules obtained from the desorption–adsorption results together with pressure vibrations.

## 5. Conclusions 

The effects of sulfuric acid-time treatment on the morphology, surface activity, and physio-chemical properties of HNTs have been investigated at four acid-time treatments of 1 h (H1), 3 h (H3), 8 h (H8), and 21 h (H21) and compared to a neat HNT (H0) sample. When sulfuric acid is added to the HNTs, the reaction of the acid with both the outer and inner surfaces of the nanotubes causes the AlO_6_ octahedral layers to dissolve, which then leads to the breakdown and collapse of the tetrahedral layers of SiO_4_. It is observed that sulfuric acid treatment breaks down the crystal structure of HNT before turning it into amorphous silica. FTIR shows hydroxyl groups were active for grafting modification by sulfuric acid, pointing to some very promising potential uses of halloysite for ceramic materials or as fillers for novel clay-polymer nanocomposites. XRD exhibits the breakdown of the crystal structure of HNTs and changes it into amorphous silica structure. In addition, the FESEM images reveal that sulfuric acid treatment dissolves the AlO_6_ octahedral layers and induces the disintegration of SiO_4_ tetrahedral layers, resulting in porous nanorods. It is also found that the BET surface area and pore volume increases when the duration of sulfuric acid treatment time is extended from 1 h to 8 h. The study has revealed very important findings regarding the type and origin of HNT used in the experiment and subsequent analyses. Seemingly, the quality and/or the source of HNT could result in significant differences regarding the behavior of treated HNT, and later in the composite that HNT is a part of it.

## Figures and Tables

**Table 1:** Results of FTIR.

Sample		H0	H1	H3	H8	H21
OH/O-H-Structure	O-H inner	3692.2	3694.7	3694.5	3694.6	-
	OH-inner	3622.23	3622.15	3622.4	3621.9	-
	O-H intramolecular	3547.68	3416.77	3402.13	3397.8	3388.02
C-C		1649.17	1648.22	1638.18	1630.5	1631.07
Si-OH		1119.13	1116.08	-	-	-
Si-O-Si		993.5	1005.72	1025.7	1031.6	1050.4
Al-OH		903.6	908.5	909.3	912.2	943.8
Al-O-OH		792.09	794.5	795.1	795.7	795.6
		748.05	751.1	751.6	-	-
Alcohol-OH-Out of Plane		-	678.0	686.6	691.25	693.35

**Table 2:** Comparison of atomic components between Zhang et al. [2] and current work.

Sample	Elements	Zhang et al. [2]		Current Work		Percentage Error	
		Weight%	Atomic%	Weight%	Atomic%	Weight%	Atomic%
H0	O	61.6	73.4	59.59	71.76	−3.31	−2.28
	Al	8.2	12.8	18.54	13.24	+55.7	+3.32
	Si	20.2	13.7	21.78	15.00	+7.25	+8.66
H8	O	60.4	72.8	60.76	72.98	≈0	≈0
	Al	9.3	6.7	6.40	4.56	−45.31	−46.92
	Si	29.5	20.2	32.84	22.46	+10.17	+10.08

**Table 3:** DSC results.

Nanotube/Treatment Time h (H)	Onset Temp. (°C)	Peak Temp. (°C)	End Temp. (°C)
Neat HNT (H0)	42.77	95.23	140.35
1 h (H1)	52.45	93.95	142.06
3 h (H3)	49.64	90.17	140.01
8 h (H8)	50.29	88.21	150.15
21 h (H21)	46.37	90.85	140.30

**Table 4:** Surface areas and pore volumes of neat HNTs and acid treatment HNTs.

Sample	H0	H1	H3	H8	H21
BET surface area (m^2^/g)	59.04	222.55	234.53	306.43	279.47
Total pore volume (cm^3^/g)	0.26	0.45	0.48	0.71	0.74
Micropore volume (cm^3^/g)	0.001	0.018	0.019	0.022	0.018
Mesopore volume (cm^3^/g)	0.28	0.49	0.52	0.81	0.82
Mesopore surface area (m^2^/g)	67.99	199.66	215.01	303.57	281.16
Average pore size (nm)	16.73	9.94	9.84	10.66	11.74
BET surface area (m^2^/g) [2]	47.8	207.6	259.1	248.4	134.1
Error% BET Surface area m^2^/g	−23.51	−7.20	+9.48	−23.34	−108.40

**Table 5:** HNT chemical composition and physical properties.

Chemical composition				
Compound	O:SiO_2_	Al:Al_2_O_3_	Si:SiO_2_	Impurities
Weight%	61.19	18.11	20.11	0.59
**Physical properties**				
Formula	Surface Area	Pore Volume	Density	Refractive Index
Al_2_Si_2_O_5_(OH)_4_·nH_2_O	65 m^2^/g	~1.25 mL/g	2540 Kg/m^3^	1.54
